# Tetramethylthiuram disulfide induces stress granules and DNA damage through oxidative stress in human lung epithelial cells

**DOI:** 10.1080/19768354.2026.2637273

**Published:** 2026-03-10

**Authors:** Ma Lin, Sangsoo Lee, Jiyun Gwak, Jihyun Cha, Seongjin Hong, Eun-Mi Kim, Kee K. Kim

**Affiliations:** a Department of Biochemistry, College of Natural Sciences, Chungnam National University, Daejeon, Republic of Korea; b Department of Earth, Environmental & Space Sciences, Chungnam National University, Daejeon, Republic of Korea; c Department of Marine Environmental Sciences & Institute of Marine Environmental Sciences, Chungnam National University, Daejeon, Republic of Korea; d Department of Bio and Environmental Technology, College of Science and Convergence Technology, Seoul Women’s University, Seoul, Republic of Korea

**Keywords:** TMTD, stress granules, oxidative stress, DNA damage, lung toxicity

## Abstract

Tetramethylthiuram disulfide (TMTD), widely used in rubber manufacturing and agriculture, presents occupational inhalation hazards, yet its effects on human lung epithelial cells remain poorly characterized. Here, we investigated TMTD-induced cellular stress responses in A549 lung epithelial cells, focusing on stress granule formation, oxidative stress, and DNA damage. TMTD induced concentration-dependent cytotoxicity, with brief exposure producing effects comparable to continuous exposure, indicating persistent cellular damage. Using live-cell imaging with A549 G3BP1-GFP knock-in cells, we demonstrated that TMTD rapidly triggered SG formation within minutes, accompanied by marked eIF2α phosphorylation. TMTD exposure caused dramatic intracellular ROS accumulation and robust γ-H2AX phosphorylation. Antioxidant rescue experiments using N-acetylcysteine confirmed that oxidative stress directly drives SG formation and DNA damage. Repeated TMTD exposure significantly increased apoptotic cell populations, demonstrating that cells cannot recover from recurrent exposure. Our findings reveal a mechanistic cascade whereby TMTD induces oxidative stress, triggers SG formation as an adaptive response, causes DNA damage, and ultimately leads to apoptosis when cellular stress overwhelms protective mechanisms. This study establishes stress granule formation as a sensitive early biomarker for TMTD exposure and highlights significant respiratory health risks for workers in rubber and agricultural industries, supporting the need for re-evaluation of occupational exposure limits and implementation of stringent protective measures.

## Introduction

Occupational and environmental exposure to chemical substances poses significant risks to human health through various routes, with inhalation being the most critical pathway in workplace settings, followed by dermal contact and ingestion (Cherrie et al. 2006). Tetramethylthiuram disulfide (TMTD), also known as thiram, is a widely utilized chemical compound in the rubber industry as a vulcanization accelerator and in agriculture as a fungicide and pesticide. While extensive research has documented TMTD's capacity to cause contact dermatitis and skin sensitization through direct exposure, the respiratory toxicity profile of this compound remains inadequately characterized despite its prevalent use in occupational environments where inhalation exposure is inevitable. We hypothesized that TMTD exposure leads to oxidative stress in lung epithelial cells, which in turn triggers stress granule formation, DNA damage, and apoptotic cell death.

TMTD is readily absorbed through the respiratory system and undergoes rapid biotransformation to dimethylthiocarbamate and carbon disulfide, metabolites that contribute to its toxic effects. Clinical case reports have documented instances of acute toxic lung injury following TMTD inhalation, with some cases progressing to respiratory failure, highlighting the urgent need for comprehensive understanding of its pulmonary toxicity mechanisms (Siwiec et al. 2019). However, most toxicological studies of TMTD have focused on animal models or plant systems, leaving a significant knowledge gap regarding its effects on human lung epithelial cells and the underlying molecular mechanisms of toxicity.

Cellular stress responses represent critical determinants of chemical toxicity outcomes. Among these responses, the formation of stress granules (SGs) has emerged as a sensitive and early indicator of cellular perturbation. SGs are dynamic, non-membranous cytoplasmic ribonucleoprotein complexes that rapidly assemble under various stress conditions to protect cellular viability by sequestering untranslated mRNAs and associated proteins (Kedersha et al. 2002; Reineke et al. 2012). Under acute stress conditions such as oxidative stress, ER stress, viral infection, and heat shock, SG formation occurs through the phosphorylation of eukaryotic initiation factor-2α (eIF2α) by specific kinases, leading to translational inhibition and subsequent mRNA-protein aggregation (Donnelly et al. 2013). While SG formation initially serves as a cytoprotective mechanism, prolonged stress or failure to resolve these structures can contribute to cellular dysfunction and has been implicated in various pathological processes, including cancer development and neurodegeneration.

Oxidative stress represents a major trigger for SG formation and is characterized by an imbalance between the production of reactive oxygen species (ROS) and the cellular antioxidant defense capacity. ROS encompass a diverse group of highly reactive oxygen-containing molecules and free radicals that, when present in excess, can cause extensive cellular damage including lipid peroxidation, protein oxidation, and DNA modifications (Cooke et al. 2003).

DNA damage induced by oxidative stress includes base modifications such as 8-oxo-deoxyguanosine formation, single and double-strand breaks, and complex clustered lesions that collectively contribute to genomic instability and mutagenesis (Juan et al. 2021). The phosphorylation of histone H2AX (γ-H2AX) serves as a sensitive biomarker of DNA double-strand breaks and is widely used to assess genotoxic potential of chemical exposures.

Given the structural characteristics of TMTD, which contains redox-active disulfide bonds and thiol-reactive groups, we hypothesized that this compound would induce oxidative stress and trigger cellular stress responses in human lung epithelial cells. The present study was designed to investigate whether TMTD exposure disrupts cellular homeostasis through the induction of SG formation, and to examine the potential for TMTD to cause DNA damage and oxidative stress. By elucidating these interconnected stress response pathways, we aim to provide mechanistic insights into TMTD-induced cytotoxicity and contribute to a more comprehensive understanding of its potential health risks in occupational and environmental exposure scenarios.

## Materials and methods

### Cell culture and chemicals

The human lung epithelial cell line A549 was obtained from ATCC. A549 cells were maintained in Roswell Park Memorial Institute 1640 (RPMI 1640; Corning, NY, USA; Cat# 10–040-CV) supplemented with 10% fetal bovine serum (Gibco, Waltham, MA, USA; Cat# 12483–020) and 1% penicillin/streptomycin (WELGENE, Gyeongsan, Korea). The cells were incubated at 37 °C; in a humidified atmosphere containing 5% CO2. TMTD (also known as Tetramethylthiuram disulfide; Bis(dimethylthiocarbamoyl) disulfide; Bis(dimethylthiocarbamyl) disulfide; Thiram), N-acetylcysteine (NAC) and sodium arsenite were purchased from Sigma-Aldrich (St. Louis, MO, USA).

### Cell viability analysis

To evaluate the effect of TMTD on cell viability, the CellTiter 96® AQueous One Solution Cell Proliferation Assay (Promega, Madison, WI, USA) was used. A549 cells were cultured in 96-well plates and treated with indicated concentrations of TMTD for 24 h or 1 h, then replaced with complete medium and recovered for 24 h. MTS reagent was added, and absorbance was measured at 490 nm using a microplate reader (Molecular Devices ABS Plus, San Jose, CA, USA). Cell viability was calculated using the following formula:

Cellviability(%)={[A(sample)–A(blank)]/[A(control)–A(blank)}]×100

### Immunoblot analysis

Whole-cell extracts were prepared with M-PER™ Mammalian Protein Extraction Reagent (Thermo Scientific, Waltham, MA, USA, Cat# 78501) supplemented with a protease inhibitor cocktail (Roche Applied Science, Basel, Switzerland). Protein concentrations were determined using a Bradford assay. Equal amounts of protein were denatured with SDS sample buffer containing β-mercaptoethanol and proteins were resolved by SDS – polyacrylamide gel electrophoresis. The resolved proteins were then transferred onto a nitrocellulose membrane (Pall Life Science, Port Washington, NY, USA). The membranes were blocked with 5% (w/v) non-fat dry milk (Rockland Immunochemicals, West Grove, PA, USA) dissolved in PBS-T supplemented with 0.05% Tween-20 for 1 h at 25 °C, and then incubated overnight with the primary antibodies at 4 °C. After washing three times with PBS-T, the membranes were incubated with horseradish peroxidase-conjugated secondary antibodies (Cell Signaling Technology, Danvers, MA, USA) at 25 °C for 1 h. Proteins were detected using a ChemiDoc MP Imaging System (Bio-Rad, U.S.). The primary antibodies used in this experiment were anti-eIF2α (1:500, Santa Cruz Biotechnology, Dallas, TX, USA; Cat# sc-133132), anti-phospho-eIF2α (1:1000, Cell Signaling Technology; Cat# 3597), anti-GAPDH (1:5000, Biodesign, Memphis, TN, USA; Cat# H86504M), and anti-γH2AX (1:1000, Abcam, Cambridge, UK; Cat# ab11174). Immunoblotting bands were quantified using ImageJ software (NIH, Bethesda, MD, USA).

### Immunofluorescence and live cell imaging

A549 G3BP1-GFP knock-in (KI) cell lines were treated with TMTD for 1 h to induce stress granule (SG) formation. The cells were then fixed in 4% paraformaldehyde in PBS for 10 min, followed by permeabilization with PBS containing 0.5% Triton X-100 (Sigma-Aldrich) for 15 min. Cells were blocked by incubating with 5% goat serum and 0.1% bovine serum albumin in PBS for 1 h followed by overnight incubation at 4 °C; with primary antibodies. The primary antibodies used were anti-G3BP1 (1:500, Santa Cruz Biotechnology, Dallas, TX, USA; Cat# sc-365338), and anti-GFP (1:500, MBL International Corporation, Woburn, MA, USA, Cat# 598) antibodies. After washing with PBS-T, secondary antibodies Alexa Fluor 488 – and 594-conjugated goat antibodies (1:2000, Thermo Fisher Scientific, Waltham, MA, USA; Cat# A11008 and A11005) against rabbit and mouse IgG were applied for 1 h at 25 °C. Cell nuclei were stained with DAPI (1:1000, Thermo Fisher Scientific; Cat# D1306). Coverslips were then washed with PBS-T and mounted using ProLong Gold anti-fade mounting medium (Thermo Fisher Scientific). Fluorescence images were acquired using a fluorescence microscope (MEIJITECHNO pE-300 lite, Tokyo, Japan).

For live cell imaging, cells were treated with TMTD at 37 ˚C in a humidified atmosphere with 5% CO2. Fluorescence images were acquired using a fluorescence microscope (MEIJITECHNO pE-300 lite, Tokyo, Japan). SG-positive cells were quantified using ImageJ software (NIH, Bethesda, MD, USA).

### SG quantification

To determine the percentage of cells containing SGs, we performed immunofluorescence experiments at 20× magnification, selecting three areas of 31684 µm². For the cell imaging experiments, three areas of 17161 µm² were selected. The ratio of SG-positive cells was quantified using ImageJ by counting cells containing at least three discrete cytoplasmic G3BP1-GFP positive foci with an area of at least 0.1 μm^2^ and an intensity at least 2-fold higher than the diffuse cytoplasmic background (Cho et al. 2024). For each condition, cells were analyzed in three randomly selected fields, with a minimum of 20 cells counted per field, and the proportion of SG-positive cells to the total number of cells within each field was calculated and expressed as a percentage. using ImageJ software. In each of the three independent areas, at least 20 cells were counted for each condition.

### Measurement of intracellular ROS levels

To measure intracellular ROS levels, two methods were employed the ROS-Glo™ H₂O₂ Assay and DCF-DA fluorescence assay: For the ROS-Glo™ H₂O₂ Assay, cells were seeded in 96-well plates (3 × 10³ cells/well) and treated according to the manufacturer’s protocol (Promega Corporation, Madison, WI, USA). The relative ROS level was calculated using the following formula:

RelativeROSlevel(%)=[RLU(sample)–RLU(control)]/RLU(control)×100.

For the DCF-DA fluorescence assay, cells seeded in 6-well plates (1.0 × 10⁶ cells/well) were trypsinized, suspended in HBSS (Welgene), and incubated with 2 μM 2,7-dichlorofluorescin diacetate (DCF-DA, Sigma-Aldrich) in HBSS at 37 °C for 30 min (Cho et al. 2024). DCF-DA fluorescence from 5,000 cells was measured using a BD FACSCanto II (BD Biosciences, Franklin Lakes, NJ, USA), and the data were analyzed with FlowJo software (BD Biosciences).

### Annexin V/PI staining

Cell death was assessed using the Annexin V Apoptosis Detection Kit (Bio-Techne, Minneapolis, MN, USA) according to the manufacturer’s instructions. Briefly, cells were harvested, washed with PBS, and resuspended in binding buffer. The cell suspensions were then incubated with Annexin V and PI for 15 min at 25 °C in the dark. Cell death rates (%) were determined using a BD FACSCanto II flow cytometer (BD Biosciences, Franklin Lakes, NJ, USA) and FlowJo™ software (BD Biosciences).

### Statistical analysis

Data are presented as the mean ± standard deviation (SD). The statistical analysis was performed using a two-tailed Student’s t-test or one-way ANOVA with Tukey’s multiple comparisons post-hoc test. Differences were considered statistically significant at *p* < 0.05.

## Results

### Exposure to TMTD reduces lung epithelial cell viability

**Figure 1.**
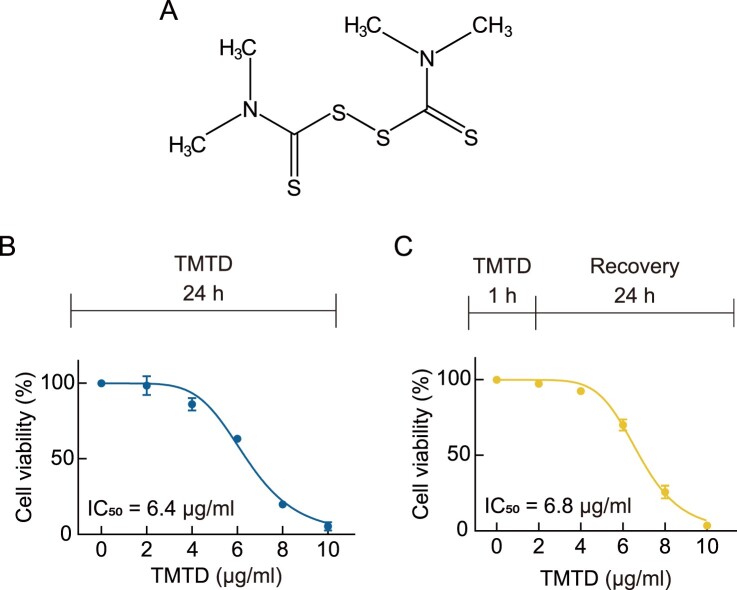
**Effect of TMTD exposure on cell viability.** (A) The chemical structure of TMTD. (B) A549 cells were treated with indicated concentrations of TMTD for 24 h, followed by MTS assay. The IC_50_ value was identified as 6.4 μg/mL. (C) A549 cells were treated with indicated concentrations of TMTD for 1 h, then replaced with complete media and recovered for 24 h, followed by MTS assay. The IC50 value was identified as 6.8 μg/mL. Results are expressed as the mean ± standard deviation (*n* = 3).

TMTD (tetramethylthiuram disulfide) is composed of two N,N-dimethylthiocarbamoyl groups connected by a central disulfide bond (Figure 1A). The disulfide linkage represents a redox-active site that can contribute to oxidative stress, while the thiocarbonyl groups are capable of interacting with cellular thiols. These structural features suggest a potential mechanism by which TMTD interferes with cellular redox balance and induces cytotoxic effects. To compare the cytotoxic effects of 24-hour and 1-hour TMTD exposure on A549 cells, cell viability was assessed using the MTS assay. Following 24-hour exposure to TMTD, cell viability remained unaffected at concentrations up to 4 μg/mL. However, at higher concentrations, a significant decrease in cell viability was observed, with an IC50 value determined to be 6.4 μg/mL (Figure 1B). When cells were exposed to TMTD for 1 h followed by 24 h of recovery, cell viability was similarly unaffected at concentrations up to 4 μg/mL. At higher concentrations, however, a significant decrease in cell viability was observed, with an IC50 value of 6.8 μg/mL, which was comparable to that of 24-hour continuous exposure (Figure 1C). These results indicate that even after 24 h of recovery following TMTD exposure, A549 cells exhibited similar viability patterns to those observed with continuous 24-hour TMTD exposure, suggesting that 1-hour exposure is sufficient to induce acute cytotoxicity.

### TMTD exposure induces stress granules (SG) formation

**Figure 2.**
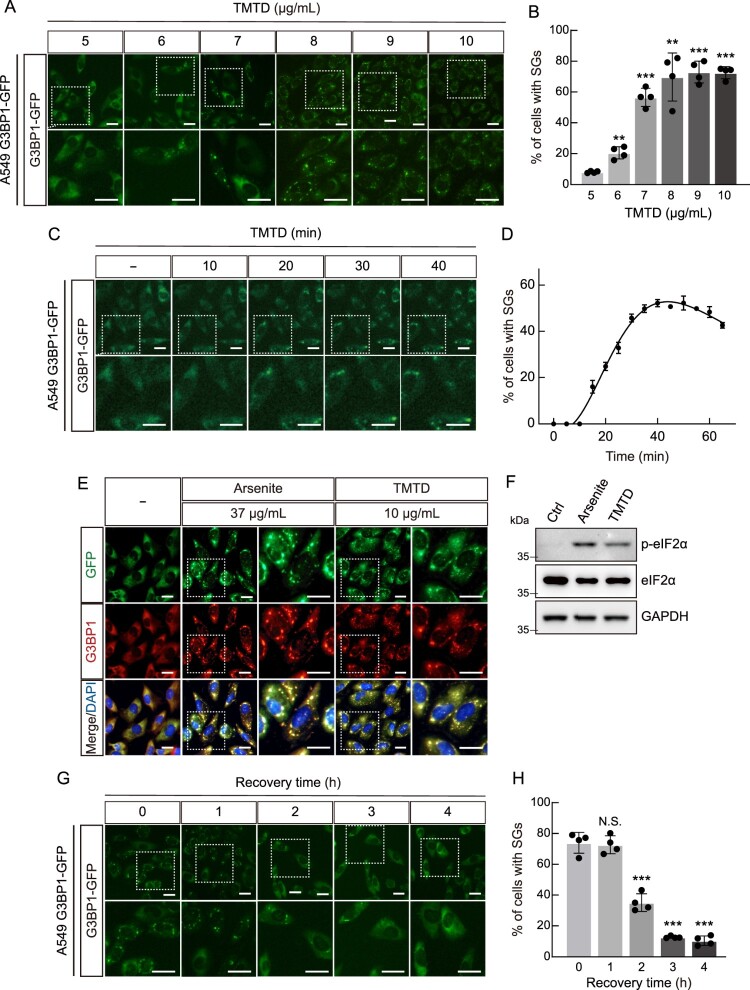
**TMTD exposure induces Stress granule (SG) formation.** (A) The formation of SGs in A549 G3BP1-GFP knock-in cells after treatment with various concentrations of TMTD. (B) The graph shows the quantification of SGs-positive cells as percentage in TMTD-treated cells. (C) The formation of SGs in A549 G3BP1-GFP knock-in cells after treatment with 10 μg/ml TMTD for 40 min. (D) The graph shows the quantification of SG-positive cells as percentage in TMTD-treated cells. (E) A549 G3BP1-GFP knock-in cells were treated with 37 μg/mL arsenite and 10 μg/mL TMTD for 40 min, followed by immunofluorescence analysis. GFP (green); G3BP1 (red). A magnification of the white box region is shown on the right. Nuclei were DAPI-stained. (F) A549 cells were treated with 10 μg/mL of TMTD for 40 min, and cell lysates were immunoblotted using the indicated antibodies. GAPDH served as a loading control. (G) SG formation in A549 G3BP1-GFP knock-in cells at different recovery times after treatment with 10 μg/mL TMTD for 40 min. (H) The graph shows the quantification of SG-positive cells as percentage in TMTD-treated cells. A magnification of the white box region is shown below. Results are expressed as the mean ± standard deviation (*n =* 4). ***p < 0.01, ***p < 0.001, N.S., not significant. Statistical comparisons between groups were performed using two–tailed Student's t-test. All scale bars, 20 μm.

Cells exposed to various stresses, including chemical exposure, activate defense mechanisms such as SG formation in order to survive (Takahashi et al. 2013). Based on our observation that 1-h exposure to TMTD cell viability significantly decreased at concentrations above 4 μg/mL, we investigated the effect of TMTD exposure on SG formation using live cell imaging. For this purpose, we utilized A549-G3BP1-GFP cells, which are A549 cells engineered using CRISPR/Cas9-mediated gene editing to express G3BP1 fused with green fluorescent protein (GFP), thereby enabling real-time investigation of SG formation and dynamics. This endogenous fluorescent SG reporter protein-based monitoring system can overcome the limitations of existing technologies and can be used for rapid and efficient toxicity assessment of various hazardous substances as described previously (Lee et al. 2024). First, A549-G3BP1-GFP cells were exposed to different concentrations of TMTD for 40 min, and SG formation was monitored by live-cell imaging. The results showed that little to no SG formation was observed at 5 μg/mL, whereas robust SG assembly was induced at 6 and 7 μg/mL TMTD. Overall, SG formation exhibited a clear concentration-dependent increase, with marked cytotoxicity observed at 6 μg/mL of TMTD (Figure 2A, B). To further investigate the temporal dynamics of TMTD-induced SG formation, A549-G3BP1-GFP cells were exposed to TMTD (10 μg/mL) for varying durations, and SG formation was monitored by live cell imaging. The formation of SGs in cells progressively increased for up to 40 min and reached a peak at 40 min (Figure 2 C,D and Video S1). To further validate these findings, SG formation following TMTD exposure was additionally examined using immunofluorescence microscopy. As expected, sodium arsenite treatment, a known SG inducer, effectively induced SG formation. A549 cells exposed to 10 μg/mL TMTD for 40 minutes exhibited G3BP1-GFP aggregation by both immunofluorescence and immunoblot analysis (Figure 2E, F). To assess the reversibility of TMTD-induced SG formation, we performed recovery experiments by removing TMTD after 40 min of treatment and monitoring SG dynamics at the indicated time points. Cells were exposed to 10 μg/mL TMTD for 1 hour, washed extensively with fresh medium, and monitored by live-cell imaging at 1-h intervals for 4 hours. SG-positive cells decreased from 74% after washing to 72.7% at 1 hour, 35% at 2 hours, 12.6% at 3 hours, and 10.3% at 4 hours post-washout (Figure 2G, H), demonstrating that SG formation is partially reversible upon stressor removal, although complete resolution requires several hours.

### TMTD exposure induces eIF2α phosphorylation

**Figure 3.**
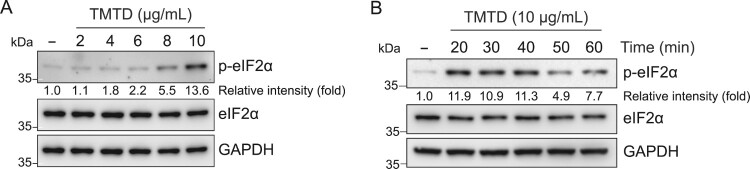
**TMTD exposure induces eIF2α phosphorylation.** (A) A549 cells were treated with indicated concentrations of TMTD for 1 h, and cell lysates were immunoblotted using the indicated antibodies. (B) A549 cells were treated with 10 μg/mL of TMTD for indicated times, and cell lysates were immunoblotted using the indicated antibodies. The values under the blot indicate the relative intensities of the phospho-eIF2α (p-eIF2α) bands. Bands were quantified and normalized to eIF2α. GAPDH served as a loading control. Band intensities were quantified using ImageJ software.

To determine whether TMTD exposure induces eIF2α phosphorylation, immunoblot analysis was performed using an anti-phospho-eIF2α antibody after treating cells with the indicated concentrations of TMTD for 40 min. Phosphorylation of eIF2α increased by 1.1-, 1.8-, 2.2-, 5.5-, and 13.6-fold in cells exposed to 2, 4, 6, 8, and 10 μg/mL TMTD, respectively (Figure 3A). To examine the temporal dynamics of TMTD-induced eIF2α phosphorylation, cells were treated with 10 μg/mL TMTD for the indicated times and analyzed by immunoblotting with an anti-phospho-eIF2α antibody. The results revealed that phosphorylation of eIF2α markedly increased between 20 and 40 min, reaching 11.9-, 10.9-, and 11.3-fold, respectively. After 40 min, phosphorylation of eIF2α gradually declined to 4.9 – and 7.7-fold at 50 and 60 min, respectively (Figure 3B). These results indicate that the cellular stress response to TMTD is rapidly triggered within 40 min and begins to recover after 40 min in a dynamic manner. Collectively, these results demonstrate that TMTD exposure induces eIF2α phosphorylation in both dose – and time-dependent manners.

### TMTD exposure induces SG and DNA damage through oxidative stress

**Figure 4.**
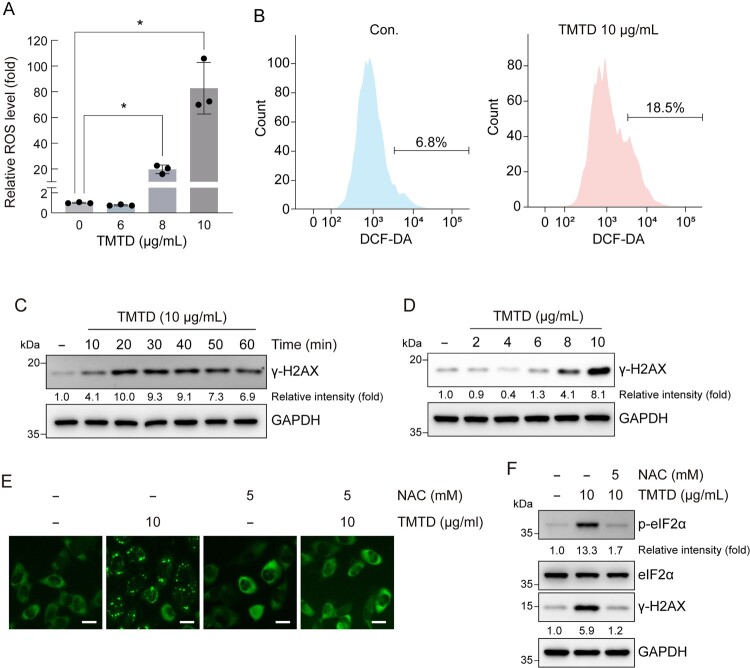
**TMTD exposure induces ROS and DNA damage.** (A) A549 cells were treated with various concentrations of TMTD for 24 h and treated with ROS-Glo™ H2O2 Assay. (B) A549 cells were treated with 10 μg/mL of TMTD for 1 h, stained with DCF-DA, and analyzed by flow cytometry. (C) A549 cells were treated with 10 μg/mL of TMTD for indicated times (D) A549 cells were treated with various concentrations of TMTD for 1 h, and cell lysates were immunoblotted using the γindicated antibodies. (E) The formation of SGs in A549 G3BP1-GFP knock-in cells pretreatment with 5mM NAC for 2 h and treatment with 10 μg/mL TMTD and 5 mM NAC for 40 min is shown. Scale bar, 20 μm. (F) A549 cells were pretreated with 5mM NAC for 2 h and treated with 10 μg/mL TMTD and 5 mM NAC for 40 min and cell lysates were immunoblotted using the phospho-eIF2α (p-eIF2α) antibody and γ-H2AX antibody. The values under the blot indicate the relative intensities of the γ-H2AX bands and phospho-eIF2α bands normalized to GAPDH. GAPDH served as a loading control. Band intensities were quantified using ImageJ software. Results are expressed as the mean ± standard deviation (*n* = 3). *p < 0.05. Statistical comparisons between groups were performed using one-way ANOVA with Tukey’s multiple comparisons post-hoc test. Scale bar, 20 μm.

To evaluate the effect of TMTD exposure on intracellular ROS levels, A549 cells were treated with the indicated concentrations of TMTD, and ROS levels were measured using the ROS-Glo H₂O₂ Assay and DCF-DA fluorescence assay. After treatment with TMTD for 24 h, ROS levels were measured using the ROS-Glo H₂O₂ Assay. The results showed that at 8 μg/mL TMTD, ROS levels increased to 19.7-fold, and at 10 μg/mL, ROS levels reached 82.7-fold (Figure 4A). These findings indicate that TMTD induces ROS accumulation in A549 cells in a concentration-dependent manner. To further confirm these observations, flow cytometric analysis using DCF-DA staining showed that after 1-hour treatment with 10 μg/mL TMTD, the proportion of ROS-positive cells increased from 6.8% in the control group to 18.5% (Figure 4B). Collectively, these results demonstrate that exposure to TMTD potently induces oxidative stress.

DNA damage has severe consequences for cell survival, and is typically followed by the phosphorylation of H2AX (Garcia-Canton et al. 2012). Therefore, to examine whether exposure to TMTD induces DNA damage, cells were treated with 10 μg/mL TMTD for the indicated times. The results revealed that γ-H2AX levels increased at 10 and 20 min, reaching 4.1 – and 10.0-fold compared to the control group, respectively. After 30 min, γ-H2AX expression gradually declined, reaching 9.3-, 9.1-, 7.3-, and 6.9-fold compared to the control (Figure 4C). These results indicate that TMTD induces a rapid and transient DNA damage response in A549 cells, with peak γ-H2AX activation occurring at approximately 20 min after treatment. Furthermore, to investigate the concentration-dependent effect of TMTD on DNA damage, cells were treated with the indicated concentrations of TMTD for 60 minutes, and immunoblotting was performed using an anti-γ-H2AX antibody. The results revealed that γ-H2AX expression increased in a concentration-dependent manner with TMTD exposure (Figure 4D). To establish a relationship between TMTD-induced ROS generation and SG formation as well as DNA damage, cells were treated with the ROS scavenger N-acetylcysteine (NAC). Following 2-hours of NAC pre-treatment, TMTD-induced SG assembly, eIF2α phosphorylation, and γ-H2AX levels were significantly suppressed, confirming that oxidative stress mediates these cellular responses (Figure 4E, F). Collectively, these results demonstrate that TMTD exposure induces oxidative stress and DNA damage in both time- and dose-dependent manners.

### Repeated exposure to TMTD induces apoptotic cell death

**Figure 5.**
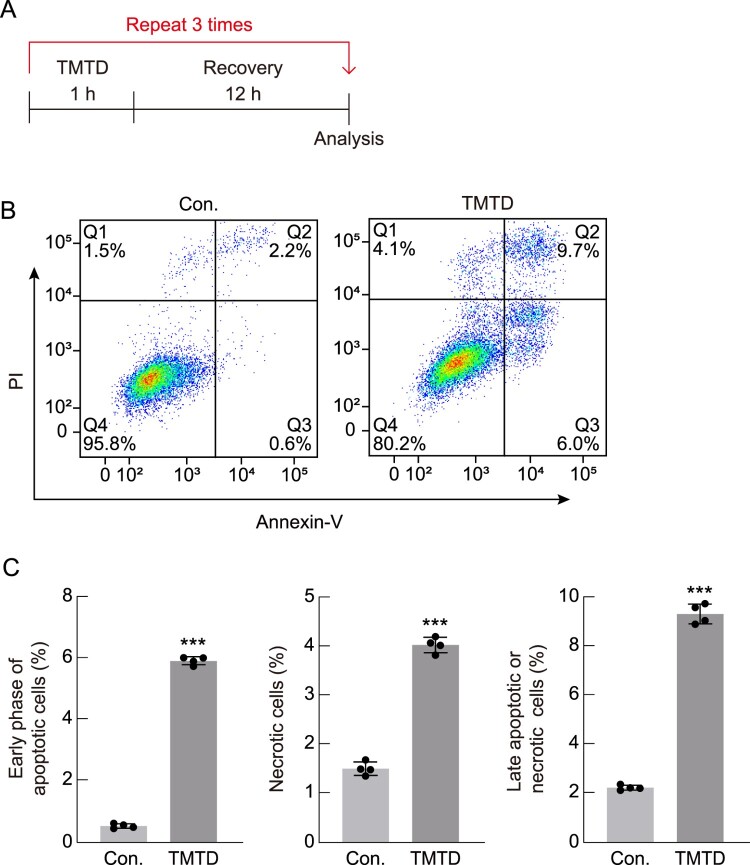
**Repeated exposure to TMTD induces cell death.** (A) Experimental scheme for repeated exposure of TMTD is indicated. A549 cells were treated with 6 μg/mL of TMTD for 1 h every 12 h and incubated. (B) A549 cells were treated three times for 1 h every 12 h intervals with TMTD, stained with fluorescein isothiocyanate-labeled Annexin V (Annexin V-FITC) and propidium iodide (PI), and analyzed by flow cytometry. Normal cells (Q4 region: Annexin V-FITC – negative/PI-negative), early apoptotic cells (Q3 region: Annexin V-FITC-positive/PI-negative), late apoptotic or necrotic cells (Q2 region: Annexin V-FITC-positive/PI-positive), and necrotic cells (Q1 region: Annexin V-FITC-negative/PI-positive). (C) The graph below quantifies early and late apoptotic cells and necrotic cells (*n* = 4). Results are expressed as the mean ± standard deviation. ***p < 0.001. Statistical comparisons between groups were performed using two–tailed Student's t-test.

To investigate the effects of repeated exposure to TMTD on A549 cells, cells were exposed to 6 μg/mL TMTD for 1 h, followed by a 12-hour recovery period over three cycles (Figure 5A). To evaluate the effect of repeated TMTD exposure on apoptosis in A549 cells, we employed Annexin V-FITC/PI double staining, as Annexin V-FITC/PI staining serves as a reliable indicator of cellular apoptosis. Annexin V selectively binds to exposed phosphatidylserine, making it useful for detecting apoptotic cells, while dual staining with Annexin V-FITC and PI allows the identification of normal cells (Q4 region: Annexin V-FITC-negative/PI-negative), early apoptotic cells (Q3 region: Annexin V-FITC-positive/PI-negative), late apoptotic or necrotic cells (Q2 region: Annexin V-FITC-positive/PI-positive), and necrotic cells (Q1 region: Annexin V-FITC-negative/PI-positive). Flow cytometric analysis revealed that when cells were repeatedly exposed to TMTD, the proportions of Annexin V-FITC-positive/PI-negative cells and Annexin V-FITC-positive/PI-positive cells significantly increased (Figure 5B,C). Collectively**,** these findings demonstrate that repeated exposure to TMTD induces severe cellular stress, ultimately causing apoptotic cell death.

## Discussion

This study provides the first comprehensive characterization of TMTD-induced cellular stress responses in human lung epithelial cells, revealing a coordinated cascade involving SG formation, oxidative stress, DNA damage, and apoptosis. Our findings demonstrate that TMTD, a widely used rubber vulcanization accelerator and agricultural fungicide, poses significant cytotoxic risks to respiratory health. The dose–response analysis revealed concentration-dependent cytotoxicity in A549 cells. Notably, even brief exposures followed by recovery periods produced cytotoxic effects comparable to continuous exposure, suggesting that TMTD triggers persistent cellular damage within a short exposure window.

A central finding is that TMTD rapidly induces SG formation through eIF2α phosphorylation, with SG formation occurring within minutes of exposure. While SG formation initially serves a cytoprotective role, our temporal analysis showing sustained SG presence and eventual apoptosis with repeated exposure suggests that the cellular stress response transitions from protective to pathological. Consistent with this, a previous study demonstrated that SG formation can serve as an early biomarker that predicts eventual cell death. Furthermore, a recent study revealed that persistent SG assembly contributes to dysregulated protein translation and defective cellular responses in various pulmonary pathologies (Ivanov et al. 2019; Hofmann et al. 2021). Consequently, these observations suggest that SG formation could serve as an early and sensitive indicator of TMTD exposure.

The oxidative stress response constitutes a key mechanistic link between TMTD exposure and cellular dysfunction, with our results demonstrating dramatic concentration-dependent ROS accumulation. The redox-active disulfide bond in TMTD's chemical structure likely contributes to ROS generation through disruption of cellular thiol-disulfide balance, mitochondrial dysfunction, and antioxidant depletion (Le et al. 2025). This oxidative stress directly drives the DNA damage observed in our study, evidenced by robust γ-H2AX phosphorylation that increased rapidly following exposure. The temporal correlation between ROS accumulation and DNA damage suggests oxidative stress as the primary driver of genotoxicity. DNA double-strand breaks can lead to chromosomal aberrations, mutations, and cell death if repair processes are overwhelmed (Jackson and Bartek 2009). And the lung epithelium is particularly vulnerable due to continuous exposure to atmospheric oxygen and environmental oxidants. Accumulating evidence links oxidative DNA damage to chronic respiratory diseases including COPD, asthma, and lung cancer, raising concerns about potential long-term health consequences of occupational TMTD exposure (Neofytou et al. 2012). We further demonstrated that SG formation is driven by oxidative stress. Further investigation of other stress-responsive pathways, including heat shock protein expression and autophagy-related markers, would provide more comprehensive insights into the cellular stress-response cascade triggered by TMTD. Such studies could elucidate how protective and maladaptive pathways are differentially activated under acute versus prolonged stress conditions, thereby clarifying the mechanisms by which cells transition from adaptive responses to irreversible dysfunction.

The demonstration that repeated TMTD exposure induces apoptotic cell death provides critical insights into the compound's chronic toxicity profile. Our experimental design involving repeated cycles of exposure and recovery was intended to simulate intermittent occupational exposure patterns, and the significant increases in both early and late apoptotic cell populations indicate that cells cannot effectively recover from recurrent TMTD exposure. While apoptosis serves as a protective mechanism, excessive apoptosis of lung epithelial cells can compromise the epithelial barrier, impair mucociliary clearance, and promote inflammatory responses, processes implicated in various pulmonary disorders (Raby et al. 2023). The integration of our findings suggests a mechanistic pathway whereby TMTD induces oxidative stress and DNA damage, triggers stress granule formation as an adaptive response, and ultimately leads to apoptosis when cellular stress becomes overwhelming. Our recovery experiments indicate that TMTD-induced SGs are reversible upon removal of the stressor. However, SG resolution represents recovery of translational control and does not necessarily reflect complete cellular recovery. Importantly, oxidative stress–induced DNA damage may persist beyond SG disassembly, potentially affecting long-term cellular outcomes. Therefore, reversible SG dynamics should not be equated with complete cellular recovery. Given that workers in rubber manufacturing and agriculture potentially experience repeated inhalation exposure to TMTD, our findings support the need for stringent exposure controls and protective measures in industries using this compound. While direct conversion between air concentrations and in vitro doses is complex, our experimental concentrations fall within a physiologically relevant range for acute exposure scenarios. This is particularly significant considering that the OSHA permissible exposure limit for TMTD is 5 mg/m³ (8-hour TWA), and peak local concentrations in the lung epithelial lining fluid during overexposure incidents may exceed this level (OSHA. 2024). Our comprehensive dose-response analysis across multiple endpoints allows us to establish the No Observed Adverse Effect Level (NOAEL) at <2 μg/mL and the Lowest Observed Adverse Effect Level (LOAEL) for early stress markers at 2–4 μg/mL. The threshold for significant cellular dysfunction was identified at 5–6 μg/mL, immediately preceding the IC50 of 6.4–6.8 μg/mL.

In addition to direct inhalation exposure, the potential for secondary effects associated with TMTD warrants careful consideration. Following its extensive industrial use, TMTD can migrate and accumulate in surrounding environments (Kupiainen et al. 2005; Zhang et al. 2018) Aquatic systems serve as the ultimate sink for many anthropogenic contaminants, with sediments representing a major repository (Ahrens et al. 2009). A previous study has reported the detection of TMTD in sediments from Lake Sihwa, a highly industrialized area in South Korea (Gwak et al. 2025), suggesting that this compound can persist in the environment without complete removal. Once deposited, such substances may be resuspended into the water column and subsequently accumulate in aquatic organisms such as fish. These contaminants can negatively affect the physiological functions of organisms and, given that fish constitute a part of the human diet, may also lead to indirect human exposure and secondary health impacts. Furthermore, not only TMTD but also other rubber additives have been reported to exert toxic effects on organisms. For instance, N-isopropyl-N′-phenyl-p-phenylenediamine-quinone, a transformation product of a tire antioxidant, has been identified as a potent toxicant causing acute mortality in Coho Salmon (Oncorhynchus kisutch) populations (Tian et al. 2021). These findings highlight that rubber additive-derived compounds, closely linked to human activities, can exert persistent and adverse ecological impacts that extend beyond simple cellular toxicity. Therefore, strengthened management and regulatory control over the use and environmental release of such compounds are urgently needed.

Several limitations warrant consideration. Our investigation utilized a single immortalized cell line under in vitro conditions, which may not fully recapitulate the complexity of human lung tissue. While we identified oxidative stress and DNA damage as key mechanisms, other cellular stress pathways including ER stress, autophagy, and inflammatory signaling may also contribute to TMTD toxicity. Future studies should employ more physiologically relevant models such as primary cells or organoid cultures, and in vivo studies are essential to evaluate systemic toxicity and long-term outcomes. Investigation of protective strategies, including antioxidant supplementation, would provide valuable information for developing interventions to mitigate TMTD-induced toxicity. In conclusion, this study establishes that TMTD induces a coordinated sequence of cellular stress responses in human lung epithelial cells, providing mechanistic insights into its respiratory toxicity and highlighting this compound as a potential hazard for workers in rubber manufacturing and agricultural industries. Our results call for re-evaluation of current exposure limits and establish SG formation as a sensitive early biomarker for exposure assessment. The molecular pathways identified here may also inform understanding of other thiol-reactive industrial chemicals.

## Author contribution

Ma Lin: Methodology; Investigation; Writing – Original Draft; Visualization, Sangsoo Lee: Investigation; Visualization, Jiyun Gwak: Formal analysis, Investigation, Jihyun Cha: Formal analysis, Investigation, Seongjin Hong: Conceptualization; Review & Editing; Supervision; Project administration, Eun-Mi Kim: Review & Editing; Supervision; Funding acquisition, Kee K. Kim: Conceptualization; Writing – Original Draft; Writing – Review & Editing; Supervision; Project administration; Funding acquisition

## Supplementary Material

Supplemental Material
